# Assessment of Cross-Reactive Neutralizing Antibodies Induction Against H5N1 Clade 2.3.4.4b by Prior Seasonal Influenza Immunization in Retail Workers

**DOI:** 10.1093/ofid/ofaf463

**Published:** 2025-08-20

**Authors:** Andrea Arroyave, Henintsoa Rabezanahary, Aude Wantchecon, Vonintsoa Lalaina Rahajamanana, Ahmed Sahli, Mathieu Thériault, Denis Boudreau, Caroline Gilbert, Sylvie Trottier, Mariana Baz

**Affiliations:** Department of Microbiology, Infectious Disease and Immunology, Faculty of Medicine, Université Laval, Quebec, Quebec, Canada; Division of Infectious and Immune Diseases, CHU de Québec Research Center, Quebec, Quebec, Canada; Department of Microbiology, Infectious Disease and Immunology, Faculty of Medicine, Université Laval, Quebec, Quebec, Canada; Division of Infectious and Immune Diseases, CHU de Québec Research Center, Quebec, Quebec, Canada; Department of Microbiology, Infectious Disease and Immunology, Faculty of Medicine, Université Laval, Quebec, Quebec, Canada; Division of Infectious and Immune Diseases, CHU de Québec Research Center, Quebec, Quebec, Canada; Department of Microbiology, Infectious Disease and Immunology, Faculty of Medicine, Université Laval, Quebec, Quebec, Canada; Division of Infectious and Immune Diseases, CHU de Québec Research Center, Quebec, Quebec, Canada; Department of Microbiology, Infectious Disease and Immunology, Faculty of Medicine, Université Laval, Quebec, Quebec, Canada; Division of Infectious and Immune Diseases, CHU de Québec Research Center, Quebec, Quebec, Canada; Department of Microbiology, Infectious Disease and Immunology, Faculty of Medicine, Université Laval, Quebec, Quebec, Canada; Division of Infectious and Immune Diseases, CHU de Québec Research Center, Quebec, Quebec, Canada; Département de Chimie et Centre d’optique, Photonique et laser (COPL), Université Laval, Quebec, Quebec, Canada; Department of Microbiology, Infectious Disease and Immunology, Faculty of Medicine, Université Laval, Quebec, Quebec, Canada; Division of Infectious and Immune Diseases, CHU de Québec Research Center, Quebec, Quebec, Canada; Department of Microbiology, Infectious Disease and Immunology, Faculty of Medicine, Université Laval, Quebec, Quebec, Canada; Division of Infectious and Immune Diseases, CHU de Québec Research Center, Quebec, Quebec, Canada; Department of Microbiology, Infectious Disease and Immunology, Faculty of Medicine, Université Laval, Quebec, Quebec, Canada; Division of Infectious and Immune Diseases, CHU de Québec Research Center, Quebec, Quebec, Canada

**Keywords:** cross-reactivity, H5N1, immunity, influenza, neutralization

## Abstract

This study evaluated whether immunity from seasonal influenza induces cross-reactive antibodies against recent avian and bovine H5N1 strains in serum from retail workers. Despite strong neutralizing activity against seasonal strains, no cross-reactivity against H5N1 was detected, emphasizing the need to assess broader immune responses and targeted vaccines for H5N1 viruses.

Since its emergence in 1997, highly pathogenic avian influenza (HPAI) H5N1 has caused widespread outbreaks in birds, resulting in significant economic losses on farms and the death or culling of over 557 million birds globally between 2005 and 2023 [1]. Sporadic human infections with H5N1 have also been reported following contact with infected animals, with over 950 documented cases worldwide and a fatality rate approaching 50% as of 2024 [2].

Following its initial detection, the H5N1 subtype evolved into multiple clades due to the diversification of hemagglutinin (HA) and neuraminidase (NA) genes [3]. Among these, the clade 2.3.4.4b was first identified in domestic poultry in Asia in 2020, primarily affecting avian species [4]. This clade exhibited a remarkable ability for geographic expansion and host adaptation, leading to its spread across multiple continents. It was detected in wild birds in the United States in 2021 and spread rapidly across North America [5].

HPAI H5N1 clade 2.3.4.4b has raised global concern due to its rapid spread, severe impact, and capacity for interspecies transmission. Spillover events have been reported in more than 50 species, raising concerns about their zoonotic potential [6]. In early 2024, H5N1 clade 2.3.4.4b (genotype B3.13) was detected for the first time in dairy cattle in the United States, along with associated human infections, with clinical presentations ranging from conjunctivitis, mild respiratory, and systemic symptoms [7, 8].

Human infections with clade 2.3.4.4b viruses from exposures to infected animals can present with a broad spectrum of clinical severity, progressing to severe and fatal pneumonia, and complications [9]. In November 2024, Canada reported its first human case of H5N1 clade 2.3.4.4b (genotype D1.1) in a teenager from British Columbia who required intensive care, with no definitive exposure source identified [10]. A few months later, the D1.1 genotype, related to viruses detected in wild birds and poultry, was also identified as the cause of the first avian influenza-related fatality in the United States recorded in Louisiana, linked to exposure to backyard flocks [11].

Due to the increasing frequency of spillovers and the enhanced zoonotic potential of clade 2.3.4.4b viruses, understanding population-level immunity is critical to assess pandemic risk and guide public health strategies [12]. Although current seasonal influenza vaccines do not confer protection against avian influenza subtypes, they remain a cornerstone of prevention efforts. These vaccines are recommended to limit the risk of coinfections, which could potentially facilitate viral reassortment and induce interhuman transmission or exacerbate disease severity [13].

Recent studies have suggested that preexisting immunity from prior infections or vaccinations against seasonal influenza may elicit cross-reactive immune responses, such as antibodies or memory T cells, and modulate the clinical course of infection with emerging influenza strains [14]. This immune background may partially explain the mild symptoms observed in some reported H5N1 human infections [15, 16].

However, the extent of cross-reactivity between seasonal immunity strains and circulating H5N1 variants remains unclear. Given its historically high pathogenicity and associated global burden [17], this poses a critical public health challenge, particularly in high-risk environments such as farms and live markets, where continued exposure may drive the emergence of new variants with increased zoonotic and pandemic potential [18, 19].

Therefore, this study evaluated whether preexisting immunity from seasonal influenza infection and/or vaccination influences the immune response to currently circulating H5N1 viruses. We evaluated neutralizing antibody activity against five seasonal influenza strains and the cross-reactive response to 2 recently isolated H5N1 subtypes of avian and bovine origin, in serum samples collected from 194 retail workers.

## METHODS

### Study Participants and Samples

This study included 194 serum samples from a cohort of participants derived from a previous study in retail workers, in which volunteer adults were recruited at the Centre Hospitalier Universitaire de Québec-Université Laval (CHUL) in Québec City between October 2021 and May 2022 [20]. All participants provided written informed consent, and information regarding participant characteristics, symptoms of respiratory infections, and vaccination status was collected (Table 1).

**Table 1.** Participant characteristics and influenza vaccination status

	Overall Study Population	
	*N* = 194	%
Age (years), mean ± SD	44 ± 15.6	…
Age groups		
18–59	157	80.9
60–75	37	19.1
Sex		
Female	108	55.7
Male	86	44.3
Influenza vaccination		
Vaccinated	45	23.2
Vaccinated in 2021–2022^a^	8	…
Vaccinated in 2021–2022 and usually^b^	22	…
Usually vaccinated^b^	15	…
Unvaccinated	149	76.8
Comorbidities		
Hypertension	30	15.5
Diabetes mellitus	14	7.2
Asthma	16	8.3
Chronic pulmonary disease	5	2.6
Cardiovascular disease	6	3.1
Liver disease	2	1.0
Cancer	9	4.6
Blood disorder	1	0.5
Immunosuppression	7	3.6
Chronic neurological disorder	5	2.6
Hypothyroidism	10	5.2
Other^c^	48	24.7

^a^Influenza vaccine only during the 2021–2022 season.

^b^Regularly received the seasonal influenza vaccine in the previous influenza seasons.

^c^Includes chronic inflammatory diseases (arthritis, Crohn's and Raynaud’s diseases, multiple sclerosis), allergies, and dyslipidemias.

### Cells and Viruses

Seven seasonal influenza strains were tested, representing the major circulating lineages of influenza A (H1N1 and H3N2) and influenza B (Yamagata and Victoria). These strains were selected based on their epidemiological relevance, the availability of validated antigens and sera, and alignment with World Health Organization and national vaccine strain recommendations of the last 5 years.

The A/California/04/2009 (H1N1)pdm09, B/Phuket/3073/2013 (B/Yamagata lineage)-like, B/Austria/1359417/2021 (B/Victoria lineage)-like, A/Darwin/9/2021 (H3N2)-like, and A/Victoria/4897/2022 (H1N1)pdm09-like influenza strains used in the serological assays were obtained from the National Institute for Biological Standards and Control and propagated in Madin–Darby canine kidney cells overexpressing the α2,6 sialic acid receptor (ST6-GalI-MDCK cells), kindly provided by Y. Kawaoka from the University of Wisconsin, Madison, Wisconsin, United States.

A/bovine/Texas/98638/2024 and A/British_Columbia/2032/2024 (H5N1) strains were obtained from the National Microbiology Laboratory (NML), Public Health Agency of Canada, and grown in Madin–Darby canine kidney cells (MDCK, ATCC CCL-34). Virus stocks were propagated and titrated in MDCK cells and then stored at −80°C until use.

The studies involving seasonal influenza strains were conducted at Biosafety Level 2 (BSL-2), and those with the highly pathogenic H5N1 strains were carried out at BSL-3 laboratories at the *CHU de Québec-Université Laval*.

### Microneutralization Assay

Live microneutralization (MN) assays to evaluate virus-neutralizing antibodies (NtAbs) were performed in MCDK cells, as described in previous studies [21]. Positive and negative controls were included in each experiment. NtAb titer was defined as the reciprocal of the serum dilution that completely neutralized the infectivity of 100 TCID_50_ of each influenza virus strain, by the absence of cytopathic effect on MDCK, as previously described.

### Statistical Analysis

A titer of 10 was assigned to serum samples with undetectable (<20) NtAbs for mean titer calculations and statistical comparisons. Quantitative variables are described by their geometric mean, standard deviation, and range. The significance of differences in NtAb titers among the 7 influenza virus strains was assessed using the Kruskal–Wallis 1-way ANOVA, followed by Dunn's multiple comparison test, with analysis performed in GraphPad Prism 10 (GraphPad Software, Inc., San Diego, California). A *P*-value of <.05 was considered statistically significant.

## RESULTS

**Figure 1.**
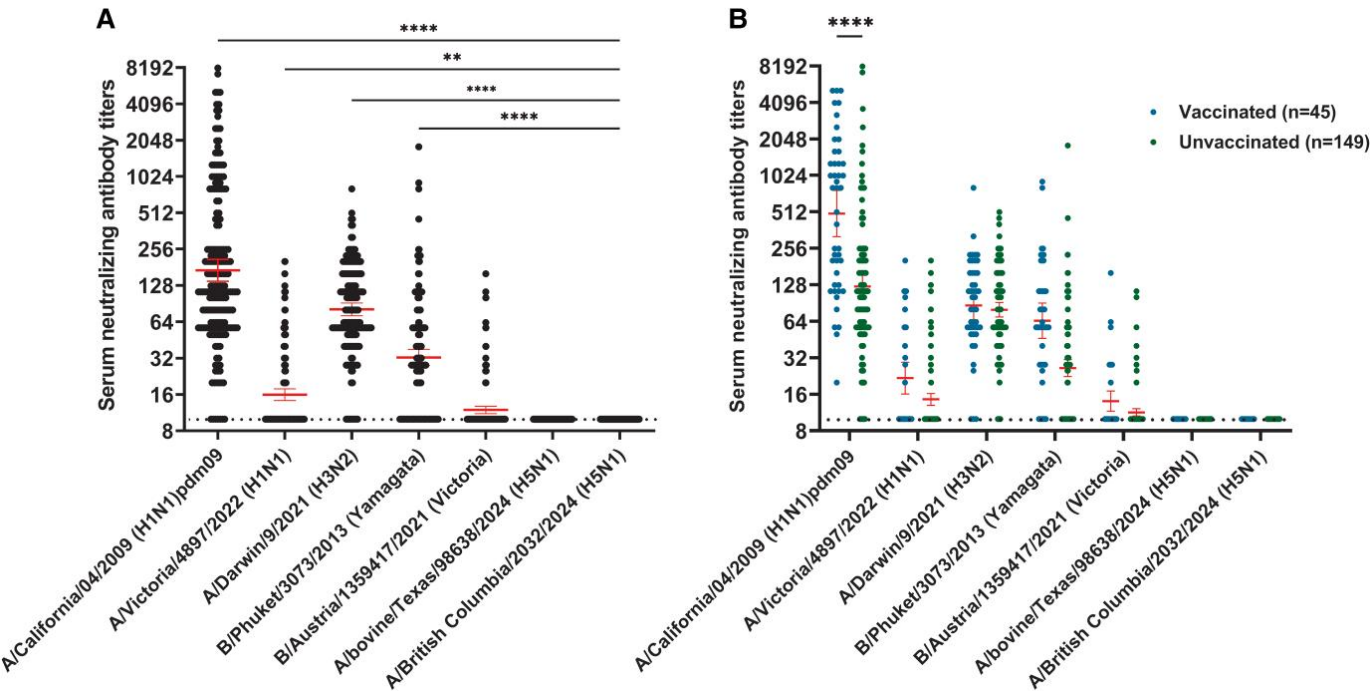
Serum neutralizing antibody titers against seasonal and avian influenza strains. (*A*) Serum NtAbs titers of all participants against influenza virus strains, including seasonal (H1N1, H3N2, and B) and avian (H5N1) strains. Each dot represents a single participant. Horizontal red bars indicate the geometric mean titers (GMTs) of neutralizing antibodies (NtAbs) in the group, with 95% confidence intervals. The horizontal dashed line represents the lower limit of detection for the microneutralization assay (neutralizing titer of 10). Statistical significance was assessed with Kruskal–Wallis 1-way ANOVA, followed by Dunn's multiple comparison test (**P ≤ .01, ****P ≤ .0001). (*B*) Serum NtAbs titers by vaccination status. Blue dots in the left represent vaccinated individuals, and green dots in the right represent unvaccinated individuals. Horizontal bars indicate GMTs. Statistical significance is shown. NtAbs: neutralizing antibodies, GMTs: geometric mean titers, MN: microneutralization.

Significant neutralizing responses were observed for seasonal influenza viruses, with geometric mean titers (GMTs) against A/California/04/2009 (H1N1)pdm09, A/Victoria/4897/2022 (H1N1), A/Darwin/9/2021 (H3N2), B/Phuket/3073/2013 (B/Yamagata), and B/Austria/1359417/2021 (B/Victoria) of 171 (range 10–8127), 16 (10–202), 81 (10–806), 32 (10–1810), and 12 (10–160), respectively. However, no measurable neutralizing activity was observed in the cohort against the H5N1 strains, A/bovine/Texas/98638/2024 and A/British_Columbia/2032/2024 (Figure 1*A*).

Participants were stratified by vaccination status. Although neither group showed detectable neutralizing activity against H5N1, vaccinated individuals tended to have higher levels of NtAbs against seasonal influenza strains compared to unvaccinated individuals, with a significant difference observed only for the A(H1N1)pdm09 virus (Figure 1*B*).

## DISCUSSION

In this study, we evaluated the NtAb responses against seasonal influenza viruses and the potential cross-reactive NtAbs against recently emerged avian influenza viruses in the Unite States and Canada in a unique cohort of participants.

Robust neutralizing activity was observed against circulating seasonal influenza strains, with titers varying across subtypes. Notably, the influenza A(H1N1)pdm09 virus elicited the highest NtAb titers, reflecting its continuous circulation and inclusion in seasonal influenza vaccines since 2009 [22]. When stratified by vaccination status, vaccinated individuals generally exhibited higher neutralizing antibody levels; however, this difference reached statistical significance only for the A(H1N1)pdm09 virus. These findings align with previous studies indicating that prior influenza exposure, whether by natural infection or vaccination, enhances immune responses against seasonal strains [23].

Conversely, no measurable NtAbs against the A/bovine/Texas/98638/2024 and A/British_Columbia/2032/2024 H5N1 strains were detected, indicating a lack of cross-reactive humoral immunity to these HPAI viruses. This result aligns with prior studies reporting limited or nonexistent serological protection against H5N1, with undetectable NtAb titers to the H5 HA protein in different populations [5, 24]. While HA proteins of H5N1 strains have shown significant antigenic and genetic variability [25], the NA proteins of the A/H5N1 viruses of clade 2.3.4.4b showed 89.6% amino acid identity with A(H1N1)pdm09 viruses, suggesting a considerable conservation of antigenic sites [5]. This raises the possibility that preexisting immunity to H1N1 may confer partial protection via cross-reactive responses to conserved NA epitopes [26].

While our results indicate limited humoral protection against H5N1, it is important to consider other immune mechanisms, such as cellular immunity, which may contribute to protection against severe disease [14]. T-cell responses induced by seasonal influenza vaccination have been shown to exhibit cross-reactivity with avian influenza strains, potentially modulating disease severity even in the absence of neutralizing antibodies [27]. However, the specific humoral and/or cellular immune responses contributing to a protective immune signature against emerging H5N1 strains remain to be fully defined.

In conclusion, our findings highlight the absence of cross-NtAb responses to 2 recently emerged H5N1 strains, despite robust immunity to seasonal influenza. Although seasonal vaccination enhances protection against currently circulating strains, it appears insufficient to elicit cross-antibody responses against antigenically distant viruses. These results underscore the need for the development of broadly protective influenza vaccines [28] and the continuous surveillance of antigenic evolution in H5N1 viruses as a public health priority [19].

Importantly, licensed H5N1 vaccines are currently available for outbreak response, with reported seroconversion rates of 60%–95% against circulating HPAI H5N1 clade 2.3.4.4b viruses [29]. However, their efficacy against emerging variants remains uncertain, and they may serve as bridging vaccines during early outbreak stages until strain-matched formulations are developed and deployed [30]. In this context, our findings reinforce the importance of proactively vaccinating high-risk groups, including individuals with occupational exposure to potentially infected animals and laboratory workers handling live H5N1 virus, identified by the National Advisory Committee on Immunization, to prevent zoonotic infections and limit opportunities for viral adaptation and transmission [29].
